# How to Ensure Inclusivity in Large-Scale General Population Cohort Studies? Lessons Learned with Regard to Including and Assessing Sex, Gender, and Sexual Orientation

**DOI:** 10.1007/s10508-023-02600-y

**Published:** 2023-04-25

**Authors:** Aranka V. Ballering, Sarah M. Burke, Els L. M. Maeckelberghe, Judith G. M. Rosmalen

**Affiliations:** 1 Department of Psychiatry, University Medical Center Groningen, University of Groningen, P.O. Box 30.001, 9700 RB Groningen, The Netherlands; 2 University Medical Center Groningen, Wenckebach Institute for Training and Education, University of Groningen, Groningen, The Netherlands; 3 Department of Internal Medicine, University Medical Center of Groningen, University of Groningen, Groningen, The Netherlands

**Keywords:** General population cohort studies, Gender identity, Sex, Intersex variations, Sexual orientation, Dutch Lifelines Cohort Study

## Abstract

Despite recent advances in the measurement of sex, gender, and sexual orientation in large-scale cohort studies, the three concepts are still gaining relatively little attention, may be mistakenly equated, or non-informatively operationalized. The resulting imprecise or lacking information hereon in studies is problematic, as sex, gender, and sexual orientation are important health-related factors. Omission of these concepts from general population cohort studies might dismiss participants’ identity and experiences and pushes research on sexual or gender minority populations toward purposive sampling, potentially introducing selection bias. It also reinforces the unintentional notion of irrelevance of these concepts to health research, ultimately disadvantaging sexual and gender minority populations. Similarly, a lack of uniform measures on sex, gender, and sexual orientation hampers multi-cohort studies in which data from multiple studies are combined, facilitating increased statistical power. This paper discusses the encountered pitfalls and lessons learned on including and assessing sex, gender, and sexual orientation in large-scale general population cohort studies, exemplified by the Dutch Lifelines Cohort Study. Additionally, we propose hands-on strategies on how to operationalize these concepts in an inclusive manner that is useful for large-scale general population cohort studies.

## Introduction

Although many advocates have been arguing for the inclusion of sex, gender, and sexual orientation in health research for decades, it has only been since the late 2000s that this movement gained momentum in epidemiological cohort studies (Klinge, 2008). In the slipstream of the increased attention for these concepts, we initiated an epidemiological research project to assess the associations between sex, gender, and common somatic symptoms in Lifelines. Lifelines is a large general population cohort study, with a three-generation design including over 1,67,000 participants from the North of the Netherlands. However, when embarking upon this project, we realized that our intended dataset did not include sufficient information to adequately answer our research questions. No information on participants’ gender, sex assigned at birth, or sexual orientation had been included during the data collection.

This lack of precise and valid information on sex, gender, and sexual orientation is not a stand-alone occurrence, but similar to other general population cohort studies (Westbrook & Saperstein, 2015). Two leading large-scale cohort studies, The UK Biobank and HUNT, do not register any dimension of gender, while a third large-scale registry, the Veterans Health Administration (VHA), does register self-reported, categorized gender identity, albeit not routinely for all participants (Åsvold et al., 2022; Bycroft et al., 2018; Grozdanic et al., 2022; Veterans Health Administration, 2022).^1^ These three studies or registries all derive sex from central registries, such as birth certificates. The UK Biobank complements their sex variable with genetic sex and does allow for adaptations in participants’ sex, rendering the sex variable a mix between recorded and self-reported sex. Similarly, the VHA allows for adjustment of birth sex. Sexual orientation is differently assessed in these studies, with the UK Biobank assessing lifetime number of same-sex sexual partners, while HUNT and VHA assesses self-reported sexual identity.

These examples align with the recent evaluation of the National Academies of Science, Engineering, and Medicine (2022) of sex, gender identity, and sexual orientation measures in research, administrative, and clinical contexts:

This evaluation revealed not only how much progress has been made in the development and refinement of sex, gender identity and sexual orientation measures that identify sexual and gender minority populations, but also how much progress remains to be made. Although measures […] become more widely implemented in data collection efforts, few of the measures in use are explicitly inclusive of gender identities that lie outside of the gender binary and many continue to rely on terminology or language that is considered invalidating or offensive to some sexual and gender minorities. (p. ix)

Thus, although increasing attention has been directed toward including and assessing sex, gender, and sexual orientation inclusively over the past decades, many leading large-scale cohort studies still use insufficient measures for these concepts. Furthermore, the lack of uniform measures on sex, gender (identity), and sexual orientation hampers multi-cohort studies on these concepts, in which data derived from multiple cohort studies can be combined to facilitate increased statistical power.

The paucity of information about participants’ sex, gender, and sexual orientation in general population cohorts is problematic, as over time a growing body of evidence has shown that these variables are important factors in health and disease (Bränström et al., 2019; Regitz-Zagrosek, 2012). Some health problems, for example, occur more frequently in women than in men, either largely due to their biological sex (e.g., breast cancer), or due to an interaction between sex and gender, for example in osteoarthrosis in which both hormonal levels and occupational hazards play a role (Laitner et al., 2021). Additionally, the literature shows that the transgender and gender diverse (TGD) population or people with a lesbian, gay, or bisexual (LGB) sexual orientation are more at risk for chronic somatic diseases and psychiatric disorders (e.g., because of minority stress and related (mental) health problems (Salomaa & Matsick, 2019)). Omission of sex, gender, and sexual orientation in studies also reinforces the unintentional notion of irrelevance of these concepts to health research.

To fully grasp the necessity to include sex, gender, and sexual orientation in health research, it is important to clarify the differences between the concepts. For example, both sex and gender have been regarded for a long time as dichotomous, synonymic concepts that can function as a proxy measure for each other, despite the two being different concepts (National Academies of Sciences, Engineering, and Medicine, 2022). Similarly, sexual orientation is a concept that is distinctly different from sex and gender (Fausto-Sterling, 2019). Therefore, we provide extensive definitions of the three concepts and their concomitant dimensions in Table 1.

**Table 1:** Definitions and dimensions of sex, gender, and sexual orientation

Sex assigned at birth and intersex variations	A biological construct that encompasses the biology, among others genes, hormones, physiology, and anatomy, of female and male bodies. Sex is usually assigned at birth. Although sex is often seen as a female/male binary, sex characteristics exist on a continuum, thus challenging the dichotomous beliefs about biological sex (Johnson et al., 2009) Intersex variations include a wide range of innate differences that relate to gonads, chromosomes, and genitals that do not fit the typical medical or social binary norms for female and male bodies (Rosenwohl-Mack et al., 2020). Strict definitions of intersex variations include chromosomal variations of the sex chromosomes (e.g., Klinefelter syndrome), genetic mutation(s) resulting in hormonal disturbances that affect sexual development (e.g., androgen insensitivity syndrome), or variations of the internal and/or external genital organs (e.g., Mayer–Rokitansky–Küster–Hauser syndrome) (Blackless et al., 2000). A more liberal definition of intersex variations also includes common variations of external genital organs, such as hypospadias or cryptorchidism for which an operation was required
Gender	The embodiment of different roles, behaviors, identities, and relationships of men and women prescribed by societal norms, which results in different expectations, opportunities, and experiences of men and women in a given society (Johnson et al., 2009)
	Gender comprises multiple dimensions (Johnson et al., 2009):
	1. Gender identity: Describes whether people identify themselves as women, men, non-binary, or another gender. Gender identity in itself is multidimensional, including (Burke, 2021):
	a) Felt-gender: the extent to which one experiences (in)congruence between the feeling of being a woman or a man, and one’s sex assigned at birth b) Gender contentedness: the degree of satisfaction or dysphoria one experiences with regard to their gender c) Conformity of gender expression: the degree of compliance with gender-related norms, such as expressing gender via hobbies or clothing
	2. Gender roles: The behavioral norms applied to people based on societal expectations and mores related to their gender
	3. Gender relations: Encompasses the interactions between people based on their ascribed gender
	4. Institutionalized gender: Refers to how power is distributed between genders in institutions
Sexual orientation	The American Psychological Association (2008) refers to sexual orientation as the description of people’s enduring pattern of emotional, romantic, and/or sexual attractions and preferences based on their sex and gender relative to the sex and gender of (a) potential partner(s), and people’s sense of identity based on those attractions, related behaviors, and membership in a community of others who share those attractions. Sexual orientation is multidimensional and can be approached from a sex perspective, a gender perspective, and a combined sex/gender perspective (Salomaa & Matsick, 2019; van Anders, 2015). The dimensions of sexual orientation include:
	1. Sexual identity: Refers to how people label themselves in relation to their partner’s or partners’ sex and/or gender preference
	2. Sexual behavior: Describes people’s potentially partnered, behaviors and activities
	3. Sexual attraction: Describes the sexual interests, approaches, attractions, and fantasies revolving around the sex and/or gender of the chosen or desired partner(s)

Although a fundamental variable such as participants’ sex is usually included in cohort studies, albeit sometimes inaccurately assessed, variables on any dimension of gender, including gender identity, and sexual orientation may be omitted by design in cohort studies. Possibly, researchers are unaware of these topics and the concomitant multidimensionality, or the omission could stem from the researchers’ idea of these being supposedly sensitive questions with which participants should not be confronted, as this could potentially result in reduced retention (Sell, 2017). Furthermore, knowledge on how to assess in a sensitive, yet informative manner participants’ biological sex, gender, and sexual orientation is lacking (Bränström et al., 2019).

Therefore, this paper aims to describe and discuss lessons learned regarding the inclusion and assessment of sex, gender, and sexual orientation in general population cohort studies. We will illustrate our points on inclusivity by using our own experiences with the assessment of sex, gender, and sexual orientation in Lifelines to show how these may be handled within general population cohort studies (Klijs et al., 2015; Scholtens et al., 2015). We will also propose concrete strategies to assess these concepts in cohort studies, while acknowledging that researchers are often constrained in what they can ask from participants by practicalities (i.e., costs, space, and participant burden) as well as participants’ potential concerns regarding their privacy and disclosure of sex, gender, and sexual orientation. Despite their separate discussion in the text, sex, gender, and sexual orientation are intrinsically linked and their interactional effect on health will be discussed as well. Ultimately, the to-be-discussed lessons refer to the larger, overarching concept of inclusivity in large-scale data studies. However, we are aware that cultural and social mores do not always allow for a setting in which sex, gender, and sexual orientation can be openly disclosed, researched, and discussed. Therefore, the lessons described here should be interpreted with cultural and social frames of reference in mind.

## Sex

Participants’ sex appears to be a straightforward concept at first glance. However, in the context of health research, it is more complicated than what may be initially expected. In Lifelines, for example, participants’ sex assigned at birth was derived from the municipal registry (Ballering et al., 2020). This resulted in an inconsistent operationalization of sex in two ways. First, the information provided was restricted to a female/male binary, which disregards the possibility of intersex variations. Second, for the vast majority of participants, municipally registered sex comprises sex assigned at birth. However, as of 2014, the Dutch law allows for individuals to change their sex in the municipal registry in a more accessible manner than before.^2^

Recently, also an “X” to indicate non-binary sex was introduced. Thus, for a minority of participants who changed their sex in the municipal registry (e.g., due to strong gender incongruent feelings) municipally registered sex may reflect their gender identity rather than their sex assigned at birth. However, although often conflated, conceptually, gender identity differs substantially from sex assigned at birth and should not be reduced to mere sex traits.

## Intersex Variations

Intersex variations include a wide range of innate differences that relate to gonads, chromosomes, and genitals that do not fit the typical medical or social binary norms for female and male bodies (Rosenwohl-Mack et al., 2020). The prevalence rate of intersex variations ranges from 0.05 to 1.7% in the general population (Blackless et al., 2000; Witchel, 2018). The variation in prevalence rates is reinforced as general population studies do not routinely include items that assess the presence of intersex variations and the exact definition of intersex variations remains a matter of debate (Rosenwohl-Mack et al., 2020). Additionally, not all intersex variations are readily identified at birth, but rather later in life. However, to facilitate research exploring sex- and thus intersex-related health factors, identification of participants with an intersex variation is required.

As no specific question assessing intersex variation was included in Lifelines, complementary approaches to identify participants with an intersex variation have been previously used^3^: Text fields of items assessing disorders, birth defects, and operations were searched for expressions of potential intersex variations, intersex birth variations, and gonad-related operations (Ballering et al., 2020). Upon applying a strict definition of intersex variations, a point prevalence for intersex variations of 0.05% in Lifelines was estimated, whereas a more liberal definition in which common variations of external organs such as hypospadias were included, yielded a point prevalence of 0.55% (Table 1). Ideally, this type of strategy should function merely as a complementary approach in addition to a specific intersex-identifying item in a survey.

Intersex variations have different etiologies. Some intersex variations have a sex-chromosomal-related etiology that can be detected by genetic approaches. In Lifelines, first-stage quality control procedures excluded participants’ genetic material that did not correspond with the municipally registered sex, as these were considered clerical or handling errors. This ultimately reduced the diversity of released data and resulted in a loss of information about intersex variations in Lifelines. This, as well as the relatively late or missed diagnosis of some intersex variations in general, likely caused Lifelines’ point prevalence to be an underestimation of the true prevalence. Currently, Lifelines also identifies relatives of participants in whom a genetic and municipally registered sex disconcordance occurs, and by using pedigree information and information provided by the family members about their relatives’ sex could confirm a sample mix-up. However, many cohort studies have no multiple-generation design and cannot assess pedigree information and familial relationships. Other large-scale cohort studies with a similar quality control pipeline, such as the UK Biobank project, did not exclude data derived from participants in whom genetically inferred sex based on sex chromosomes differed from self-reported sex (Bycroft et al., 2018). Rather, data derived from participants with a potential intersex variation or TGD identity were indicated as such, maintaining an inclusive and diverse study population. Some intersex variations are not readily detectable by genetic screening of the sex chromosomes as described above, and in some general population cohort studies no genetic approaches are included in the design. Therefore, expanding the male/female binary of participants’ sex with a non-binary option in the assessment is pivotal in obtaining more detailed data about people with intersex variations, allowing for more tailored research in this population. Therefore, Table 2 describes a set of survey items that allow for identification of participants with an intersex variation. People with an intersex variation may be assigned a sex at birth that reflects their sex characteristics at time of birth, which are not necessarily indicative of an intersex variation. Thus, by including “intersex” as an option when assessing participants’ sex assigned at birth, inconsistent results may be obtained. Therefore, an additional item that describes intersex variations allows for the identification of intersex people in a general population cohort (National Academies of Sciences, Engineering, and Medicine, 2022). The item in Table 2 is congruent with the current Dutch context, as of recently Dutch legislation eased the process of assigning an “X” on a birth certificate, indicating that the sex assigned at birth could not be irrefutably determined.

**Table 2:** Survey items including a non-binary/intersex option

Could you indicate your sex assigned at birth, as stated on your birth certificate?
Male (M)
Female (F)
Non-binary (X)
Other, please write down your preferred term
Were you born with a variation in sex characteristics (this is sometimes called intersex or an intersex variation)?
Yes
No

## Gender

Many cohort studies, including Lifelines, do not include specific questions assessing any dimension of participants’ gender. However, for our studies we were interested in the independent associations between gender roles and sex, and common somatic symptoms. Therefore, we recently showed how a data-driven method can be used to calculate a composite gender index based on participants’ gendered psychosocial characteristics for cohorts that lack data on gender (Ballering et al., 2020). We defined a gender score that quantified participants’ adherence to feminine and masculine psychosocial characteristics including but not limited to hobbies, personality traits, type of profession, time spend on household activities, and dietary preferences. As a result, participants were placed on a continuum ranging from 0%, i.e., fully masculine, to 100%, i.e., fully feminine.

The method is suitable for general population cohort studies that lack measures on gender and facilitates a gender measure specific to the context of the study. A strong advantage of this measure is that it is sensitive to the time, place, and society-bound nature of gender roles. Other existing measures, including the Bem Sex Role Inventory, have been criticized and are argued to hold limited validity to operationalize femininity and masculinity (Bem, 1974; Donnelly & Twenge, 2017; Lippa & Connelly, 1990). These instruments measure gender via items that stereotype masculine and feminine traits, while gender roles are a broad concept that is largely dependent on the respective time, place, and society (Ballering et al., 2020).

Gender measures based on previously collected survey data usually assess gender roles and/or gender relations and cannot capture participants’ current gender identity (Ballering et al., 2020; Pelletier et al., 2015; Smith & Koehoorn, 2013) (Table 1). Although gender roles and gender identity are intrinsically linked, a gender identity measure, in contrast to a gender role measure, cannot be calculated after data collection. Yet, ideally, both participants’ gender roles and gender identity are assessed in cohort studies as both dimensions are known to affect TGD and cisgender participants’ health substantially (Ballering et al., 2020; Johnson et al., 2009; Muilwijk et al., 2022).

Gender identity is a fluid, continuous, and multidimensional concept. The embodiment and expression of gender identity may differentiate over time, especially in adolescents (McHale et al., 2009; Westbrook & Saperstein, 2015), allowing for fluidity of gender identity to be captured by a repeated measures design. The continuous nature of the concept can be captured by assessing participants’ feminine or masculine identity on unipolar two-dimensional continuous scales. This allows for measuring the extent of participants’ adherence to gender identities. Preferably, gender identity should be assessed via at least a two-step approach, in which assessment of one’s sex assigned at birth and current gender identity are combined (Table 3). This allows for identification of participants with gender incongruent feelings.^4^

**Table 3:** Items included in Lifelines, based on the two-item approach combining sex assigned at birth and current gender identity, with quantification of missing data

	Municipally registered female participants (*N* = 31,058)	Municipally registered male participants (*N* = 21,588)
	Missing, *N* (%)	Missing, *N* (%)
Most people are born as either a man or a woman and they feel comfortable in a male or female body, respectively. However, this is not the case for everyone. Some people consider themselves a man, but were born in a female body or vice versa. Some people consider themselves neither a man nor a woman. Could you indicate which statement fits your experience best?^a^		
Could you indicate which statement fits your experience best?	104 (0.3%)	56 (0.3%)
My sex assigned at birth was female and I currently consider myself a woman		
My sex assigned at birth was male and I currently consider myself a man		
My sex assigned at birth was female and I currently consider myself a man		
My sex assigned at birth was male and I currently consider myself a woman		
Different, namely		
Some people, both men and women, consider themselves masculine, for example because they have characteristics or hobbies that most people consider masculine. On the other hand, both men and women may consider themselves feminine, because they have characteristics or hobbies that most consider feminine. Some people consider themselves neither masculine nor feminine Could you indicate, on a scale ranging from 1 to 10 in which 1 equals *strongly disagree* and 10 equals *strongly agree*, to what extent you consider yourself feminine or masculine? Please complete both questions		
I consider myself feminine	110 (0.4%)	272 (1.3%)
(1) Strongly disagree < < < < < < < < < < > > > > > > > > > > (10)		
Strongly agree		
I consider myself masculine (1) Strongly disagree < < < < < < < < < < > > > > > > > > > > (10)	338 (1.1%)	95 (0.4%)
Strongly agree		

^a^Potentially, the item could be expanded with additional answer options in which a non-binary gender identity is included

Gender identity is multidimensional and thus multiple, interlinking domains together define one’s gender identity. Building further on the initial model for the multidimensionality of gender identity (Egan & Perry, 2001), studies refined the dimensions (Burke, 2021; Potter et al., 2021). Recent studies, for example, include (1) felt-gender, (2) gender contentedness, and (3) gender conformity (Table 1) (Joel et al., 2014; Potter et al., 2021). The multidimensionality of gender identity calls for an approach that moves beyond the common two-step approach that merely combines sex assigned at birth and current gender identity. Previous studies, that assessed a dimensional approach to gender identity and gender incongruency, have proven the validity of multi-item questionnaires in both adults and adolescents (Deogracias et al., 2007; Singh et al., 2010). However, as the number of items that can be included in general population cohort studies is limited due to the supposed burden for participants, as well as space and cost considerations, including items on the multiple dimensions of gender identity may be more feasible in smaller add-on studies that have a specific focus on gender identity in relation to health.

Despite the potential stigma that may surround non-cisgender identities, it has been shown that the common two-step approach is easily understandable, well accepted, and causes little to no resistance in both cisgender and TGD participants in cohort studies (Bauer et al., 2017; Lombardi & Banik, 2016). Lifelines recently included an assessment of gender adapted from the two-step approach. This assessment includes gender identity and gender roles and was reviewed by a participant panel, including TGD participants, before implementation. Importantly, out of 52,646 adult Lifelines participants, only 0.3–1.3% of the male participants and 0.3–1.1% of female participants did not answer these questions (Table 3). This indicates that the vast majority of participants was willing to complete this item.

## Sexual Orientation

Akin to gender identity, specific questions regarding sexual orientation are frequently omitted in general population cohort studies. In Lifelines, for example, merely the binary sex of participants’ current partner is assessed. This is only an indirect measure from which participants’ sexual orientation could be inferred. If information on participants’ sex assigned at birth and current gender identity is unknown, the information obtained by this item is even more multi-interpretable.

Notably, the way in which questions on sexual orientation are phrased could influence the distribution of sexual orientation in a study sample (Savin-Williams, 2016). There is no generalizable rule about how items on sexual orientation should be phrased. Partly, this relates to the ongoing debate on the central axis around which sexual orientation revolves. Does one’s sexual orientation revolve around the partner’s sex, gender, or both? As van Anders (2015) states:

For example, if one is sexually attracted to men, is one attracted to penises? Social identities? Body frames? Interactions? And, how is sexual orientation defined if one is attracted to masculinity regardless of the sex of the person presenting or embodying it? (p. 1177)

Some theorize, however, that sexual orientation relates to additional concepts beyond potential partner’s sex and/or gender, such as partner number and partner age (van Anders, 2015).

Sexual orientation is also a multidimensional concept, with three separate dimensions: sexual identity, sexual behavior, and sexual attraction (Table 1) (Salomaa & Matsick, 2019; van Anders, 2015).^5^ The apparent relevance of asking for participants’ sexual orientation correlates directly with participants’ willingness to complete items on sexual orientation in relation to health (Brooks et al., 2018). Yet, not all dimensions of sexual orientation are relevant to assess in every setting. Whether or not it is appropriate and relevant to ask participants about a dimension of sexual orientation depends on the context and research question. For example, during a consult with their GP, patients may be more aware of their sexual behavior influencing their health, and they more readily disclose such information (Brooks et al., 2018). In this case, information about sexual orientation is of direct importance to people’s own health. Similarly, when donating blood, it is clearly explained why the survey administered during the intake asks for donor’s sexual behavior. Here, information about one’s sexual behavior may be of direct importance to transfusion safety. These examples illustrate people’s willingness to disclose information on sexual behavior, as long as the rationale for assessing it is clear to participants. In large-scale cohort studies, it is necessary as well to clearly explain the health-related relevance underlying items on sexual orientation and to explain that sexual orientation may associate with the development of both psychological and physical health conditions (Cochran et al., 2017; Sandfort et al., 2006), and that knowledge hereon is important for public health.

Many general population cohort studies assess sexual orientation by merely asking about participants’ sexual identity in terms of lesbian/gay, straight (i.e., not gay or lesbian), or bisexual, while it has been recently recommended to move beyond mere self-reported identity and to include sexual attraction and possibly behavior as well (National Academies of Sciences, Engineering, and Medicine, 2022). First, although self-reported identity measures allow for a relatively easy-to-analyze outcome measure, it may enforce oversimplified categorization of participants’ sexual orientation. Second, it cannot explicate the central axis of a participant’s sexual orientation and an asexual option is frequently overlooked. Third, such self-reported sexual identity items may cause confusion for TGD participants, as they may not know whether to reason from their sex assigned at birth or current gender identity. Even among researchers no consensus exists on whether sex assigned at birth or current gender identity should be used as reference to define sexual orientation (Guillamon et al., 2016; Lawrence, 2010), rendering sexual identity items multi-interpretable. Fourth, sexual identity (and behavior) may be strongly constrained by local mores and culture and may not fully reflect participants’ sexual orientation (e.g., in conservative religious communities). Last, sexual attraction underlies and complements behavior and identity, rather than behavior and identity underlying sexual attraction (Bailey et al., 2016).

To at least partly overcome these disadvantages of solely assessing sexual identity, we do not argue to abandon a self-reported sexual identity item in large-scale population cohort studies. We rather argue for complementing such an identity item with gynephilia and androphilia items. This provides an option for assessing sexual orientation that facilitates flexibility, yet restricts the answer options in such a way that they remain meaningful for general population cohort studies such as Lifelines (Table 4), in which currently no items assess sexual orientation adequately. We propose to complement a self-reported sexual identity item with two unidimensional scales on which participants can indicate their sexual attraction in terms of gynephilia or androphilia in general population cohort studies. By using two unipolar scales, participants’ sexual orientation can be approached and analyzed in a continuous manner, disregarding the need for categorization. It allows for participants to indicate asexuality, degree of same-sex sexual attraction, and degree of other-sex attraction.

**Table 4:** Proposed survey items on sexual orientation for general population cohort studies

1—Could you indicate on the scales below what describes you best?
I am sexually attracted to men
(1) Not at all < < < < < < < < < < > > > > > > > > > > (10) Very strongly
I am sexually attracted to women
(1) Not at all < < < < < < < < < < > > > > > > > > > > (10) Very strongly

For specific research questions related to sexual orientation and add-on studies, this item could potentially be extended by two additional unipolar scales on which participants can indicate their sexual attraction with a gender focus, instead of a sex focus. However, as the distinction between sex and gender frequently remains unclear among the general population, we propose to only assess sexual orientation with a focus on sex in general population cohorts as items with a gender focus may be misinterpreted by participants. To avoid confusion about sexual orientation in TGD or people with an intersex variation, items on sexual orientation should be combined with questions about people’s sex assigned at birth and current gender identity (Tables 2 and 3). To the best of our knowledge, assessing sexual orientation on two unipolar scales referring to gynephilia and androphilia has not been implemented in large-scale general population cohort studies. Future research is needed to validate these items and to compare their results with those obtained by items assessing self-reported, categorized sexual identity.

## Toward an Inclusive Future of Research

Here, we have described and discussed our experiences and lessons learned regarding sex, gender, and sexual orientation in large-scale general population cohort studies. We have described pitfalls in assessing and including these concepts, and we have proposed strategies to operationalize these in an inclusive manner relevant for the research question at hand.

It should be emphasized that obtaining detailed information about participants’ sex, gender, and sexual orientation in general population cohort studies is pivotal. First, disregarding these variables in general population cohort studies excludes the possibility of conducting studies within TGD and LGB subpopulations in a general population cohort, especially since the large study populations of cohort studies potentially allow for identification of a relatively large TGD and LGB subpopulation herein as well. As a result, studies focusing on TGD and LGB populations are usually pushed toward convenience and purposive sampling, potentially introducing selection bias (Savin-Williams, 2016). Ultimately, this results in a decreased external validity of study results (Salway et al., 2019). Nevertheless, selection bias cannot be fully dismissed in general population cohort studies either, as TGD and LGB populations may conceal aspects of their sex, gender, and/or sexual orientation (Hottes et al., 2016), resulting in an underrepresentation of TGD and LGB populations and non-random misclassification of sexual and gender minority populations potentially decreasing the validity of research findings. Second, excluding detailed information on sex, gender, and sexual orientation from general population cohort studies reinforces the current status quo in which sexual and gender minority populations are disadvantaged. Third, health-related research focusing specifically on TGD and LGB populations may ultimately contribute to better healthcare and health outcomes for these populations (e.g., by designing more personalized health interventions). Particularly large general population cohort studies have the potential to identify new or more complex associations between risk factors and health of sexual and gender minority populations, but this requires adequate identification of participants’ sex, gender, and sexual orientation.

Nevertheless, we acknowledge that researchers are constrained by practicalities (e.g., costs and participant burden) in what they can ask from participants: The number of items in a survey, their contents, and wording should be carefully balanced. Therefore, it follows that questions about sex, gender, and sexual orientation should be tailored to the specific setting and goal of the research (Salomaa & Matsick, 2019). Also, survey items that deviate from the sex/gender binary or heteronormative stance may cause resistance in relatively few participants (Joel et al., 2014; Morgenroth et al., 2020). On the other hand, omitting survey items that deviate from these norms may feel like a denial of participants’ identity or lived experiences to those who identify beyond these norms (Spiel et al., 2019; Suen et al., 2020).

Furthermore, purposeful omission of survey items that go beyond dichotomous sex, gender identity, and sexual orientation, or purposeful inclusion of dichotomous items, is a normative assumption in itself: Researchers should not automatically assume that participants refuse to answer these items. In contrast, recent evidence shows that participants often appreciate being able to share information about these topics (Case et al., 2006; Medeiros et al., 2020; Sell, 2017) provided that it is clear to participants that their information is handled in compliance with local institutional and legal privacy guidelines aimed at, among others, avoiding re-identification of anonymized or pseudonymized participants. We strongly feel that the rather small chance of resistance does not outweigh omission of inclusive items, if these allow researchers to assess and possibly aid in improving the health and empowerment of disadvantaged sexual and gender minority populations. However, to ensure acceptance as much as possible, survey items and explanatory notes on sex, gender, and sexual orientation should be implemented in collaboration with a diverse participant panel. Similarly, the collaboration with a diverse participant panel may allow for a reduction in participants’ potential concealment of sex, gender, and sexual orientation.

In conclusion, to ensure inclusivity in large-scale general population cohort studies, researchers and participants need to understand the relevance, but also the nuances and multidimensionality of participants’ sex, gender, and sexual orientation. Accounting for the lessons learned described here is a step toward an inclusive future of research, but to achieve optimal inclusivity, awareness about these concepts and their interconnectedness should be routinely ingrained in the design of general population cohort studies.

## Data Availability

Not applicable.

## Footnotes

1 HUNT is an acronym for the Norwegian name of the study “HelseUndersøkelsen i Nord-Trøndelag”.

2 Although it is possible to legally change one’s sex since 1985 in a Dutch municipal registry, several criteria, including proof of a gender confirming surgery, were necessary. Therefore, TGD that did not undergo surgery could not change their sex in the official registries before 2014.

3 A similar strategy as described here was used to identify TGD participants in Lifelines, as no specific question on gender identity and gender contentedness was included in Lifelines until 2020.

4 Researchers may consider to add an “() I prefer not to disclose option”. We decided here to exclude this option, as in general population cohort studies supposedly sensitive information, for example on traumatic experiences, is assessed as well, without providing an “I prefer not to disclose” option. If researchers wish to add this answer option, it should be added consistently throughout all items in the survey to avoid a reinforcement of the notion that information about sex, gender, and sexual orientation is more sensitive than other information.

5 Sexual orientation is sometimes regarded as a subdimension in itself (van Anders, 2015), equated with what we call sexual attraction in this text. For reasons of clarity we use the term sexual attraction, instead of sexual orientation, to describe the sexual interests, approaches and fantasies revolving around the sex and/or gender of one’s chosen partner(s).
